# Total error shift patterns for daily CT on rails image-guided radiotherapy to the prostate bed

**DOI:** 10.1186/1748-717X-6-142

**Published:** 2011-10-24

**Authors:** Ronaldo Cavalieri, Hiram A Gay, Jingxia Liu, Maria C Ferreira, Helvecio C Mota, Claudio H Sibata, Ron R Allison

**Affiliations:** 1 Department of Radiation Oncology, Clínicas Oncológicas Integradas, Rio de Janeiro, Brazil; 2 Department of Radiation Oncology, Washington University School of Medicine, St. Louis, MO, USA; 3 Division of Biostatistics, Washington University School of Medicine, St. Louis, MO, USA; 4 Department of Radiation Oncology, The Brody School of Medicine, East Carolina University, Greenville, NC, USA; 5 Department of Radiation Oncology, Carolinas Medical Center - Northeast, Concord, NC, USA; 6 Physics and Engineering Department, 21st Century Oncology, Fort Myers, FL, USA; 7 21st Century Oncology, Greenville, NC, USA

**Keywords:** Post-prostatectomy radiotherapy, IGRT, CT on rails, shift total error, prostate bed

## Abstract

**Background:**

To evaluate the daily total error shift patterns on post-prostatectomy patients undergoing image guided radiotherapy (IGRT) with a diagnostic quality computer tomography (CT) on rails system.

**Methods:**

A total of 17 consecutive post-prostatectomy patients receiving adjuvant or salvage IMRT using CT-on-rails IGRT were analyzed. The prostate bed's daily total error shifts were evaluated for a total of 661 CT scans.

**Results:**

In the right-left, cranial-caudal, and posterior-anterior directions, 11.5%, 9.2%, and 6.5% of the 661 scans required no position adjustments; 75.3%, 66.1%, and 56.8% required a shift of 1 - 5 mm; 11.5%, 20.9%, and 31.2% required a shift of 6 - 10 mm; and 1.7%, 3.8%, and 5.5% required a shift of more than 10 mm, respectively. There was evidence of correlation between the x and y, x and z, and y and z axes in 3, 3, and 3 of 17 patients, respectively. Univariate (ANOVA) analysis showed that the total error pattern was random in the x, y, and z axis for 10, 5, and 2 of 17 patients, respectively, and systematic for the rest. Multivariate (MANOVA) analysis showed that the (x,y), (x,z), (y,z), and (x, y, z) total error pattern was random in 5, 1, 1, and 1 of 17 patients, respectively, and systematic for the rest.

**Conclusions:**

The overall daily total error shift pattern for these 17 patients simulated with an empty bladder, and treated with CT on rails IGRT was predominantly systematic. Despite this, the temporal vector trends showed complex behaviors and unpredictable changes in magnitude and direction. These findings highlight the importance of using daily IGRT in post-prostatectomy patients.

## Background

External beam radiotherapy (RT) is an adjuvant treatment option for post-prostatectomy pT3 or margin-positive disease (R1), and a salvage option for those with a rising PSA post-operatively. In the adjuvant setting, three prospective randomized trials have shown that post-operative RT significantly reduces the risk of biochemical failure and disease recurrence in pT3 and R1 prostate cancer [1-3].

Intensity modulated radiation therapy (IMRT) is a well established technique for definitive prostate RT and can reduce acute and late toxicity [4,5]. It has been utilized for the salvage or adjuvant treatment of post-prostatectomy patients [5-7]. Compared to three-dimensional conformal radiotherapy (3D-CRT), IMRT has the capacity of lowering the dose to the bladder especially when it is full [6]. IMRT can also reduce the dose to the rectum compared to 3D-CRT [8]. Small treatment margins may be more sensitive to geometrical uncertainties, including setup deviations and internal organ motion, making geographic miss a matter of concern.

Therefore, it has become increasingly important to employ image guided radiotherapy (IGRT) to minimize the probability of missing the target and/or over treating normal tissues. Recently, a number studies have described the setup shifts and organ motion observed in post-prostatectomy patients using various imaging techniques and intervals [6,7,9-13]. Thus far the total number of post-prostatectomy patients analyzed has been small, and further studies are needed to better characterize this patient population. To our knowledge, this is the first study that describes individual daily total error shift patterns in post-prostatectomy patients from a diagnostic-quality CT-on-rails IGRT system.

## Methods

From April, 2007 to June, 2008, 17 consecutive post-prostatectomy patients were treated at our institution with IMRT to the prostate bed using a CT-on-rails IGRT system. The median age was 63 years, with a range of 58 to 77 years. The clinical characteristics of all patients are listed in Table 1. The prostate bed's isocenter daily total error shifts were evaluated for a total of 661 CT scans. The daily total error shift = total positioning error = prostate bed motion + setup error as defined by Schiffner *et al *[11].

**Table 1:** Patient characteristics

Age	
median	63
range	58 - 77
Gleason score	
6	5 (29%)
7	9 (53%)
8	2 (12%)
9	1 (6%)
PSA, median (range) ng/mL	
Pre-op	10.1 (3.7 - 46.2)
Pre-XRT	0.6 (0.1 - 2.8)
T Stage	
pT2c	6 (35%)
pT3a	4 (24%)
pT3b	7 (41%)
Radiotherapy intent	
Adjuvant	7 (41%)
Salvage	10 (59%)

The patients received RT as an adjuvant treatment within 6 months after surgery in the presence of high risk pathologic factors, or salvage therapy more than 6 months post-prostatectomy when there was evidence of biochemical failure. The median interval from surgery to RT was 3.6 months for adjuvant RT patients, and 29 months for salvage treatment patients.

The planning CT was obtained with a 3 mm slice thickness and the patient in the supine position. Patients were instructed to have an empty bladder and rectum for the planning CT. Subsequently, during the course of treatment, bladder and rectal filling was variable with no specific instructions given to the patient. The immobilization system consisted of a neck support and a leg sponge (Dual Leg Positioner, CIVCO, Kalon, IA).

The clinical target volume (CTV) was defined as the prostate bed. The prostate bed encompassed the anatomical volume of the resected prostate tissue, including surgical clips, residual seminal vesicles, vesico-urethral anastomosis, periprostatic tissue, bladder neck, and posteriorly to the rectal wall. A clinical correlation with the operative report and final pathology report were always used to assist in defining this volume. The planning target volume (PTV) was generated with a 10 mm margin around the CTV except 7 mm posteriorly to reduce the volume of irradiated rectum. The treatment isocenter was set up and at the time of CT planning approximately at the center of the CTV. Right lateral and anteroposterior digital reconstructed radiographs (DRRs) were generated to show the location of the isocenter. The patient was subsequently brought to the simulation room and under fluoroscopic guidance the DRRs' isocenter was identified. Skin triangulation points were then marked on the patient's skin as guided by the laser coordinate system of the simulator. All of the patients were prescribed a dose of 7560 cGy in 42 fractions to the PTV with a 7 field IMRT technique.

Daily pre-treatment CT images were acquired on a CT-on-rails system (CTVision™, Siemens, Malvern, PA) to visualize the prostate bed and make any necessary adjustments to ensure target coverage. The system consists of a CT scanner and a linear accelerator opposing each other in the treatment vault and sharing the patient couch. The axes were defined as follows: x-axis (negative, right; positive, left), y-axis (negative, caudal or inferior; positive, cranial or superior), and z-axis (negative, anterior; positive, posterior).

The alignment of the prostate bed consists of the following steps:

1. The patient's skin marks are aligned to the setup lasers to localize the isocenter. Radiopaque adhesive fiducials 1.5 mm in diameter (Suremark^® ^Markers™, The Suremark Company, Simi Valley, CA) are placed on the patient's skin, and the treatment couch is rotated 180 degrees to obtain the pre-treatment CT scan with a 1.5 mm slice thickness. The treatment couch is rotated back and the patient alignment is verified.

2. Using the CT on rails software and the radiopaque fiducials on the pre-treatment CT, the radiation therapist defines the location of the isocenter.

3. The radiation therapist manually fuses the bony anatomy from the planning CT with the pre-treatment CT as the initial coarse adjustment. If the therapist observes that there is a mismatch in the bony fusion due to rotation, the patient is re-positioned and re-scanned. Since the CT fusion software does not display the yaw, pitch or roll angles, the decision to reposition the patient is based on the therapist's experience.

4. Once the initial bony fusion is acceptable, the therapist makes fine adjustments by visually inspecting the location of the prostatectomy surgical clips and identifying the prostate bed between the bladder and rectum. By using these anatomical and surgical clip visual cues, the therapist further adjusts the isocenter.

5. Once the isocenter's fine-tuning is complete, the planning CT PTV is overlaid on that day's CT image to ensure adequate target coverage.

6. If there is doubt regarding the fusion, or if the x-axis, y-axis, and z-axis isocenter shift exceeds 1 cm, the patient's physician is contacted for approval or further adjustments. Once the fusion is deemed acceptable, the isocenter shift in the x-axis, y-axis, and z-axis is obtained from the software. There is no correction of rotation.

7. After the therapist adjusts the couch in the x, y and z axes, the patient is treated.

### Statistical analysis

The descriptive statistics of x-axis, y-axis and z-axis shifts were calculated. Pearson correlation coefficients were obtained between the x-axis and y-axis, x-axis and z-axis, and y-axis and z-axis daily shifts for each patient. A p value < 0.05 was taken to indicate statistical significance. A univariate analysis (ANOVA) was used to test the x-axis, y-axis and z-axis patient daily shifts, and a multivariate analysis (MANOVA) was used to test the daily shifts in the (x, y), (x, z), (y, z) and (x, y, z) axes. For the MANOVA and ANOVA analyses, a p value < 0.05 corresponded to a systematic error. The data were analyzed with the SAS statistical software (SAS Institute, Cary, NC). Shift errors were classified as random or systematic.

The average error is the average of all measurements in a given direction (LR, CC, or AP), with positive and negative values representing opposite directions as previously defined. The mean magnitude is the average of the absolute values of all the measurements in a given direction. Consequently, mean magnitudes can only be positive numbers, while average errors could be positive or negative.

## Results

**Figure 1:**
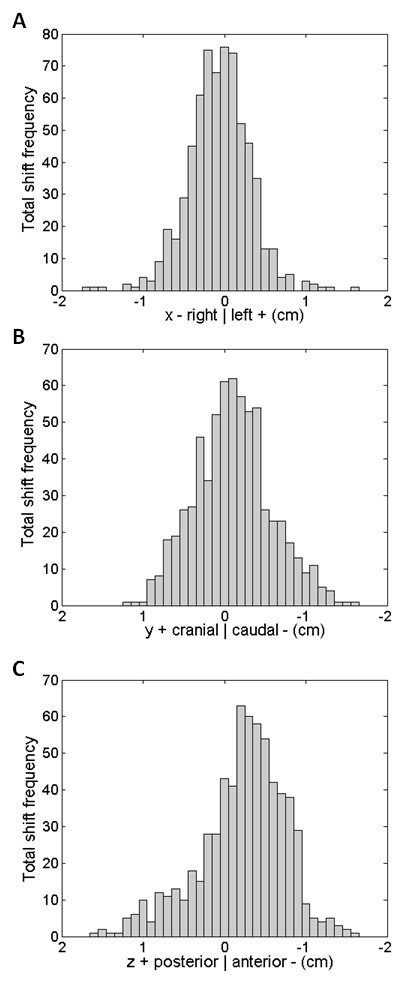
**Histograms showing the total error shift frequency in the (a) x-axis (negative, right; positive, left), (b) y-axis (negative, caudal or inferior; positive, cranial or superior), and (c) z-axis (negative, anterior; positive, posterior) for all 17 patients totaling 661 IGRT CT scans**.

A total of 661 daily CT scans were performed with an average of 39 scans per patient. The median bladder volume was 75 cc (range 35.9 - 300.7 cc). Fourteen of seventeen patients had volumes ≤ 150 cc and consistent with mostly empty bladders. In the right-left direction, 11.5% of the 661 scans required no adjustments, 75.3% required a shift of 1 - 5 mm, 11.5% required a shift of 6 - 10 mm, and 1.7% required a shift of more than 10 mm (Figure 1a). The distribution had a kurtosis of 1.74, and was negatively skewed with a skewness of -0.06. The standard deviation was 3.8 mm and the mean -0.6 mm.

In the cranial-caudal direction, 9.2% of the scans required no adjustments, 66.1% required a shift of 1 - 5 mm, 20.9% required a shift of 6 - 10 mm and 3.8% required a shift larger than 10 mm (Figure 1b). The distribution had a kurtosis of -0.02, and was negatively skewed with a skewness of 0.19. The standard deviation was 4.7 mm and the mean -1.1 mm.

Finally, in the posterior-anterior direction, 6.5% of the scans required no shifts, 56.8% required a shift of 1 - 5 mm, 31.2% required a shift of 6 - 10 mm, and 5.5% required a shift of 10 mm or more (Figure 1c). The distribution had a kurtosis of 0.52, and was positively skewed with a skewness of 0.63. The standard deviation was 5.3 mm and the mean -2.2 mm.

**Figure 2:**
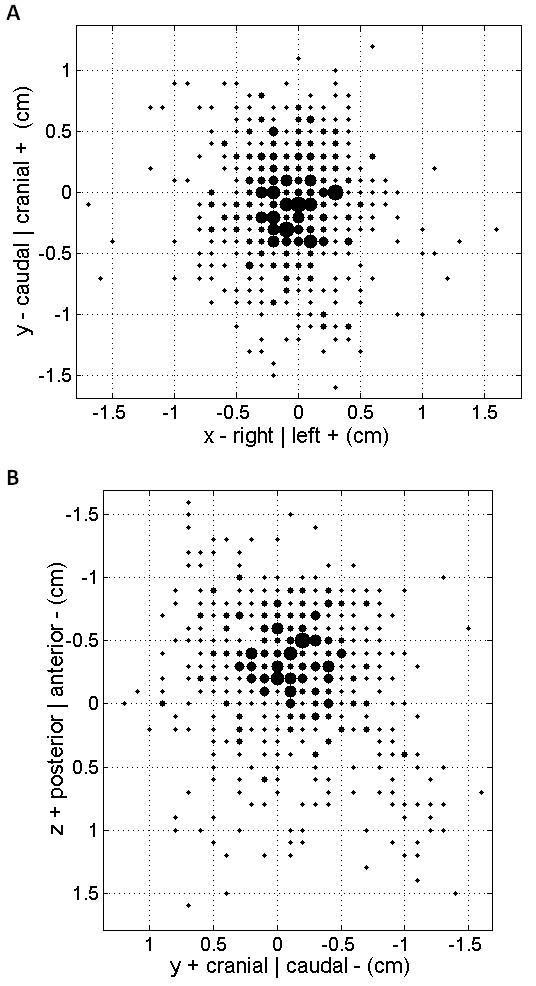
**Bubble plot of the total error combinations in (a) the y-axis and x-axis and (b) the z-axis and y-axis for all 17 patients**. The size of the bubble is proportional to the number of observations. The largest bubble represents 10 observations, while the smallest one represents 1.

**Figure 3:**
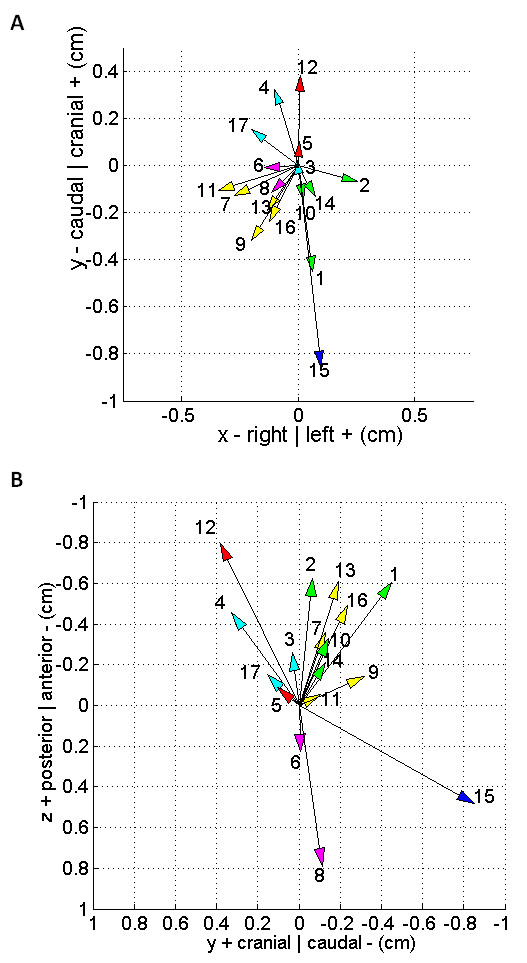
**Average total error vectors in (a) the y-axis and x-axis (anteroposterior view) and (b) the z-axis and y-axis (lateral view) for all 17 patients**. The average vectors pointed towards six of the eight possible octants. Vectors sharing the same colored arrowhead point to the same octant (for example, patients 7, 9, 11, 13, and 16). Patient numbering as in Table 2.

Figure 2 shows the frequency of the total shift combinations in the anteroposterior (Figure 2a) and lateral (Figure 2b) views. Figure 3 shows the average total shift vector for each patient. The average vectors pointed towards six of the eight possible octants. None of the patients had average vectors pointing in both the posterior and cranial direction which accounts for the two missing octants.

**Figure 4:**
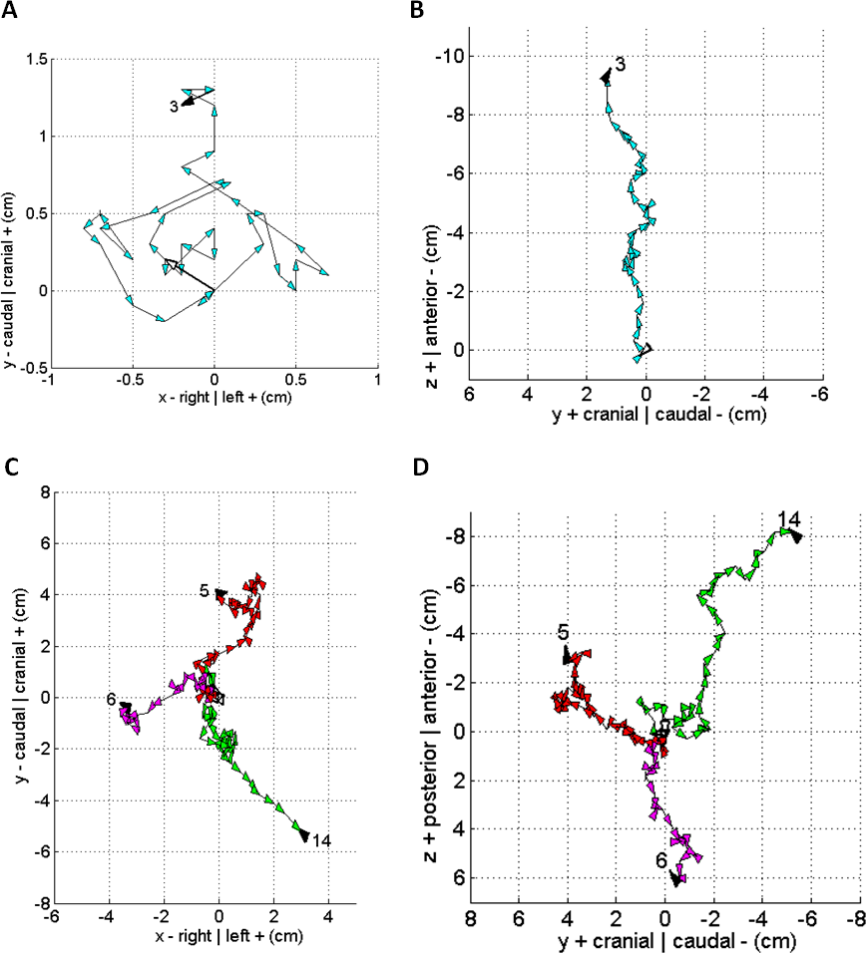
**Sequential plot of daily total error shift vectors for each patient**. The vector direction is indicated by the arrowhead which due to scaling often obscures the true magnitude of the vector. Unique vector and arrowhead color combinations were used when multiple patients were plotted in the same graph. The origin of the vector from the initial IGRT CT is always at coordinate (0,0) and has a white arrowhead. The final IGRT CT has a black arrowhead pointing to the patient number. Patient numbering as in Table 2. Patient 3's anteroposterior (a) versus lateral (b) daily vector plot. Patients 5, 6, and 14 anteroposterior (c) versus lateral (d) daily vector plot.

**Figure 5:**
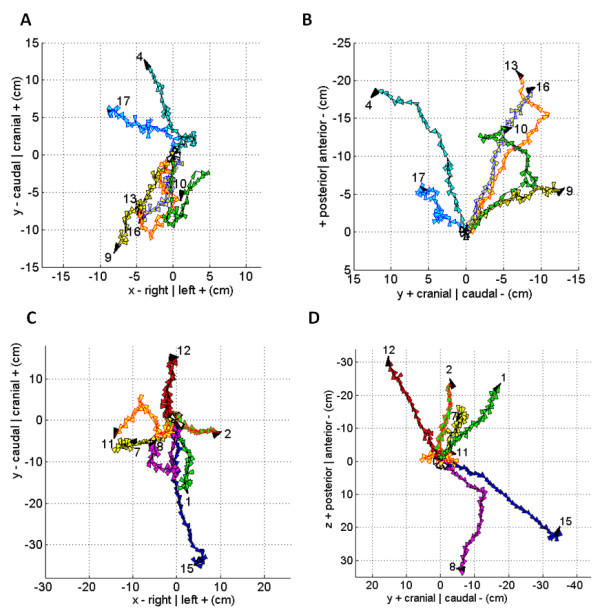
**Same description as figure 4**. Patients 4, 9, 10, 13, 16, and 17 anteroposterior (a) versus lateral (b) daily vector plot. Patients 1, 2, 7, 8, 11, 12, and 15 anteroposterior (c) versus lateral (d) daily vector plot. Note that patients were grouped in four groups generally with similar but increasing total displacement.

Figures 4 and 5 illustrate the daily shift vectors in sequential order for each patient, and roughly groups the patients in order of increasing total displacement. For example, figure 4a and 4b shows patient #3 who had the least total displacement, while figures 5c and 5d include the patients with the most total displacement or distance traveled by the vector summation.

Overall, the Pearson correlation coefficient between x-axis and y-axis was -0.05 (p = 0.17), between x-axis and y-axis was -0.06 (p = 0.11), and between y-axis and z-axis was -0.21 (p < 0.0001) respectively. At the patient level, the Pearson correlation coefficients between the x-axis and y-axis, between x-axis and y-axis, and between y-axis and z-axis are given in Table 2.

**Table 2:** Pearson correlation coefficients, ANOVA univariate, and MANOVA multivariate x, y and z axis analysis

Patient	Pearson Correlation Coefficients			ANOVA			MANOVA			
			
	x-axis vs y-axis	x-axis vs z-axis	y-axis vs z-axis	x-axis	y-axis	z-axis	x-axis, y-axis	x-axis, z-axis	y-axis, z-axis	x, y, z axis
	ρ (Pearson)	ρ (Pearson)	ρ (Pearson)	F	F	F	F	F	F	F
1	-0.19	0.27	-0.21	2.46	**83.45**	**223.96**	**40.62**	**125.06**	**186.19**	**125.67**
2	-0.07	-0.06	-0.05	19.07	**5.61**	**285.42**	**11.41**	**144.34**	**143.94**	**96.44**
3	-0.09	-0.01	-0.18	0.02	1.01	**46.48**	0.50	**22.61**	**22.62**	**14.67**
4	**-0.32**	-0.08	-0.02	3.40	**37.11**	**42.66**	**18.08**	**23.46**	**38.06**	**24.75**
5	0.22	**-0.39**	-0.28	0.03	**6.23**	2.65	3.12	1.42	**3.50**	2.47
6	0.15	-0.16	-0.38	**7.31**	0.02	**12.73**	**3.54**	**8.36**	**6.94**	**6.07**
7	0.22	0.02	**0.37**	**7.69**	**9.35**	**34.7**	**6.84**	**20.36**	**17.37**	**13.27**
8	-0.15	-0.29	0.15	2.93	1.21	**174.89**	2.35	**87.72**	**89.97**	**59.58**
9	-0.26	-0.02	0.21	**27.47**	**163.44**	**11.24**	**118.60**	**19.18**	**79.89**	**77.07**
10	**0.40**	0.07	**0.37**	0.14	2.52	**43.18**	1.82	**21.40**	**21.48**	**14.04**
11	-0.04	0.15	-0.17	**60.11**	1.31	1.39	**30.41**	**29.32**	1.59	**19.79**
12	0.04	-0.15	-0.16	0.04	**102.91**	**120.85**	**50.17**	**59.95**	**94.12**	**61.96**
13	-0.02	-0.11	-0.13	2.09	**4.66**	**113.96**	**3.32**	**58.81**	**61.48**	**42.21**
14	-0.16	0.05	-0.16	3.06	**4.48**	**11.77**	3.16	**7.54**	**9.29**	**6.75**
15	**-0.40**	**0.32**	**-0.77**	2.36	**230.63**	**29.65**	**123.99**	**14.48**	**157.84**	**109.26**
16	0.12	**-0.31**	0.04	**7.52**	**40.63**	**134.85**	**21.65**	**87.31**	**82.91**	**64.69**
17	-0.30	0.07	-0.20	**21.81**	**8.10**	**7.63**	**11.75**	**13.58**	**6.38**	**9.18**

**Bold **numbers represent statistically significant values (p < 0.05). In addition, for the ANOVA and MANOVA analyses, **bold **numbers also represent systematic errors, while non-bold numbers represent random errors.

Table 2 also shows the F statistics from the univariate analysis (ANOVA) and the F statistics from the multivariate analysis (MANOVA). The ANOVA analysis suggests that the following patients had systematic errors in the following single axes:

• x-axis: 2, 6, 7, 9, 11, 16, and 17

• y-axis: 1, 2, 4, 5, 7, 9, 12-17

• z-axis: 1-4, 6-10, 12-17

The MANOVA analysis suggests that the following patients had systematic errors in the following combination of axes:

• x and y axes: 1, 2, 4, 6, 7, 9, 11-13, 15-17

• x and z axes: 1-4, 6-17

• y and z axes: 1-10, 12-17

• x, y, and z axes: 1-4, 6-17

## Discussion

As IMRT has rapid falloff of dose distribution it has become evident that IGRT should be considered a necessity for reproducible tumor targeting and/or sparing of normal tissues. In the post-prostatectomy patient population, Simpson *et al*. did not report any toxicities greater than grade 2 when using cone-beam computed tomography (CBCT) and onboard imaging IGRT [13,14]. Ost *et al*. reported a lower incidence of grade 1 and 2 genitourinary toxicity in their CBCT group compared with their electronic portal imaging device (EPID) group [14]. These studies highlight the value of IGRT in the post-prostatectomy setting.

Other studies have used surgical clips [7,10], or gold fiducials [11] as surrogates of the prostate bed. Whether surgical clips or gold fiducials are truly representative of the entire prostate bed and can accurately reflect the prostate bed motion remains to be proven. Despite the differences in methodology, Table 3 shows that our total error results are consistent with comparable studies using either the mean magnitude or average error methods [7,11-13].

**Table 3:** Comparison of total errors observed in patients receiving postoperative radiotherapy to the prostate

			Mean magnitude (mm)			Standard deviation (mm)		
**Series**	**Modality**	**patients/images**	**LR**	**CC/(SI)**	**AP**	**LR**	**CC (SI)**	**AP**

Sandhu et al. ^7^	KV orthogonal	26/384	3.9	5.3	3.8	5.9	8.1	5.5
Ost et al. ^12^	CBCT	15/547	2.9	1.9	3.1	2.2	1.6	2.3
Simpson et al. ^13^	kV planar	27/725	3.9	3.8	4.8	2.8	1.9	3.2
	CBCT	23/752	2.8	1.8	3.2	3.5	3.7	4.4
present study	CT	17/661	2.9	3.8	4.7	2.5	3.0	3.3
								
			**Average (mm)**			**Standard deviation (mm)**		

**Series**	**Modality**	**patients/images**	**LR**	**CC/(SI)**	**AP**	**LR**	**CC (SI)**	**AP**

Schiffner et al. ^11^	EPID	10/163	0.2	1.2	-0.3	4.5	5.1	4.5
Ost et al. ^12^	CBCT	15/547	1.5	-0.5	1.7	3.3	2.4	3.4
present study	CT	17/661	-0.6	-1.1	-2.2	3.8	4.7	5.3

Negative shifts are in the right, inferior or anterior directions.

Abbreviations: LR = left-right axis; CC = cranial-caudal axis = SI = superior-inferior axis; AP = anterior-posterior axis. KV = kilovoltage, EPID = electronic portal imaging device; CBCT = cone-beam computed tomography; CT = computed tomography

The average error is the average of all measurements in a given direction. The mean magnitude is the average of the absolute values of all the measurements in a given direction.

For 11 patients the smallest total error vector displacement was in the x axis, and the rest were equally divided between the y and z axes. For 12 patients the largest total displacement was in the z-axis, for 3 it was in the y-axis and for 2 in the x-axis.

Figure 3a shows that the cumulative vectors mostly pointed in the caudal and anterior directions. This could be explained by the direction of the forces exerted by the bladder and rectum. Pinkawa *et al*. has described decreasing bowel and rectal volumes during the course of treatment from urinary and bowel urgency [6]. Similarly, constipation or urinary retention that resolve or start during the course of treatment could further alter the direction of the forces exerted by the bladder and rectum, and account for some of the pattern changes observed during the course of treatment.

None of the patients had average vectors pointing in both the cranial and posterior direction. One potential explanation is that while the patients were instructed to have an empty bladder and rectum for simulation, there was no departmental policy to tell patients to keep an empty bladder or rectum during treatment. Compared to the empty bladder and rectum planning CT, random rectal and bladder filling during treatment may have predominantly pushed the prostate bed anteriorly and/or posteriorly and caudally, respectively.

There was evidence of correlation between the x and y, x and z, and y and z axes in 3, 3, and 3 of 17 patients, respectively. Consequently, for the majority of patients there was no correlation between the axes (Table 2). Univariate (ANOVA) analysis showed that the pattern was random in the x, y, and z axis for 10, 5, and 2 of 17 patients, respectively (Table 2). Therefore, in the cranial-caudal (y-axis) and anterior-posterior (z-axis) directions systematic errors were more common, while in the right and left direction (x-axis) random errors were more common. Furthermore, multivariate (MANOVA) analysis showed that (x,y), (x,z), (y,z), and (x, y, z) errors were random in 5, 1, 1, and 1 of 17 patients, respectively (Table 2). The multivariate analysis suggests that errors were predominantly systematic.

One limitation of this study is that only the total error was analyzed, while other studies also determined the magnitude of the prostate bed motion and the setup error. Both Schiffner and Sandhu *et al*. reported that the prostate bed motion error was smaller than the setup error [7,11]. Both the setup error and prostate bed motion errors may be subject to both random and systematic components which vary from patient to patient. Other limitations are that intrafraction motion was not analyzed, the initial and subsequent daily filling of the bladder and rectum in the planning CT was not assessed, and the potential interobserver variability in performing the planning and daily CT fusion was not evaluated. Carefully documenting changes in urinary urgency, urinary retention, and diarrhea during treatment, whether these were treatment related or not, could have helped to further understand the total error patterns. Although we simulated most patients with low volumes of urine in the bladder, we recommend that patients have full bladders during simulation and treatment for better bladder sparing. How this would alter the results we obtained remains to be seen.

Figures 4 and 5 highlight how statistical analyses, average shift vectors, or making shift assumptions based on sampling a portion of the patient's treatment is flawed. Although the total error shift vectors can at times behave predictably, they can also unpredictably change their direction and magnitude during treatment. For example, figure 5d shows how patient 8 has systematic daily total error vectors pointing in one direction during the beginning of treatment followed by about a 90 degree change in direction for the rest of the treatment. In the same figure, although patient 15 has a systematic error through most of the treatment, at the end of treatment the behavior becomes random. Each vector time trend tells a unique story for each patient, highlighting the importance of daily IGRT.

Future areas of research would be to compare bony IGRT versus soft tissue IGRT, similar to what has been done for intact prostate patients [15], and study the anatomical deformation of the prostate bed.

## Conclusions

The overall daily total error shift pattern for these 17 patients simulated with an empty bladder, and treated with CT on rails IGRT was predominantly systematic. Despite this, the temporal vector trends showed complex behaviors and unpredictable changes. These findings highlight the importance of using daily IGRT in post-prostatectomy patients.

## Competing interests

The authors declare that they have no competing interests.

## Authors' contributions

RC and HAG contributed equally to this work. RC and RRA designed the study. RC gathered the clinical patient data and created Table 1. HAG created all figures and Table 3. JL performed the statistical analysis and Table 2. MCF gathered the total shift data. HCM, CHS, and the rest of the authors helped with the interpretation of the data. All authors read and approved the final manuscript.
